# Clinical analysis of chronic active EBV infection with coronary artery dilatation and a matched case–control study

**DOI:** 10.1186/s13023-021-01689-5

**Published:** 2021-01-28

**Authors:** Ang Wei, Honghao Ma, Liping Zhang, Zhigang Li, Yitong Guan, Qing Zhang, Dong Wang, Hongyun Lian, Rui Zhang, Tianyou Wang

**Affiliations:** 1 Beijing Key Laboratory of Pediatric Hematology Oncology; National Key Discipline of Pediatrics (Capital Medical University); Key Laboratory of Major Diseases in Children, Ministry of Education; Hematology Oncology Center, Beijing Children’s Hospital, Capital Medical University, National Center for Children’s Health, Beijing, 100045 People’s Republic of China; 2 Hematology and Oncology Laboratory, Beijing Pediatric Research Institute, Beijing Children’s Hospital Affiliated with Capital Medical University; National Center for Children’s Health; Beijing Key Laboratory of Pediatric Hematology Oncology, Key Laboratory of Major Diseases in Children; Ministry of Education, National Key Discipline of Pediatrics, Beijing, 100045 People’s Republic of China; 3 Department of Hematology and Oncology, Beijing Children’s Hospital, Capital Medical University, Nanlishi Road No. 56, Xicheng District, Beijing, 100045 People’s Republic of China

**Keywords:** Epstein–Barr virus, Coronary artery, Clinical characteristics, Pathogenesis

## Abstract

**Objective:**

To investigate the clinical characteristics, treatment, prognosis and risk factors for chronic active Epstein–Barr Virus infection (CAEBV) associated with coronary artery dilatation (CAD) in children.

**Methods:**

Children with CAEBV associated with CAD hospitalized at Beijing Children’s Hospital, Capital Medical University from March 2016 to December 2019 were analyzed. Children with CAEBV without CAD were selected as the control group and matched by sex, age, treatment and admission time. The clinical manifestations, laboratory and ultrasound examinations, treatment and prognosis of the children were collected in both groups.

**Results:**

There were 10 children with CAEBV combined with CAD, including 6 males and 4 females, accounting for 8.9% (10/112) of CAEBV patients in the same period, with an onset age of 6.05 (2.8–14.3) years. The median follow-up time was 20 (6–48) months. All the patients had high copies of EBV-DNA in whole blood [1.18 × 10^7^ (1.90 × 10^5^–3.96 × 10^7^) copies/mL] and plasma [1.81 × 10^4^ (1.54 × 10^3^–1.76 × 10^6^) copies/mL], and all biopsy samples (bone marrow, lymph nodes or liver) were all positive for Epstein–Barr virus-encoded small RNA. Among the 10 children, 8 had bilateral CAD, and 2 patients had unilateral CAD. After diagnosis, 7 children were treated with L-DEP chemotherapy in our hospital. After chemotherapy, four patients underwent allogeneic hematopoietic stem cell transplantation (HSCT). The others were waiting for HSCT. At the time of the last patients follow up record, the CAD had returned to normal in 3 patients, and the time from the diagnosis of CAD to recovery was 21 (18–68) days. LDH, serum ferritin, TNF-α and IL-10 levels were statistically significantly different between the two groups (*P* = 0.009, 0.008, 0.026 and 0.030). There were no significant differences in survival rate between the two groups (*P* = 0.416).

**Conclusion:**

The incidence of CAEBV with CAD was low. CAEBV with CAD did not influence the prognosis. Patients who had high LDH, serum ferritin, TNF-α, and IL-10 levels early in their illness were more likely to develop CAD.

## Introduction

Epstein–Barr virus (EBV) belongs to the gamma–herpesvirus family, which consists of double-stranded DNA viruses. The primary infection mainly invades B lymphocytes and can cause infectious mononucleosis (IM) and EBV-associated hemophagocytic lymphohistiocytosis (HLH). EBV can also occasionally infect T lymphocytes and/or natural killer (NK) cells, resulting in EBV-driven T/NK-cell lymphoproliferative diseases, such as chronic active EBV infection (CAEBV) [1, 2].

The diagnosis of CAEBV is based on clinical manifestations and evidence of EBV in tissues or peripheral blood samples. In CAEBV, EBV-infected T or NK cells clonally proliferate and infiltrate multiple organs, leading to their failure. CAEBV has two characteristics: systemic inflammation and neoplastic disease. The main clinical finding of CAEBV is inflammation, which is characterized by fever, lymphadenopathy, liver dysfunction, hepatosplenomegaly, and an abnormal hemogram [3]. CAEBV causes vasculitis due to the direct invasion of the infected cells, which can lead to the development of vascular aneurysms. However, CAEBV combined with coronary artery dilatation (CAD) has been rarely reported [4, 5]. To date, there is no unified and effective chemotherapy regimen for CAEBV. The only effective treatment strategy is allogeneic HSCT. According to Sawada’s [6] report, a 3-step strategy including allogeneic HSCT for the treatment of CAEBV was proposed. The 3-year overall survival rate (3y-OS) was 87.3 ± 4.2%. Here, we report 10 cases of pediatric patients diagnosed with CAD secondary to CAEBV.

## Patients and methods

### Patients

From March 2016 to December 2019, 10 children suffering from CAEBV combined with CAD were enrolled in this study. All these patients fulfilled the diagnostic guidelines. Data were retrospectively reviewed for the clinical manifestations, laboratory findings, age at the onset, and therapy. We performed a retrospective matched case–control study (1:2) to identify the risk factors for CAEBV combined with CAD in the pediatric population. The control subjects were patients with CAEBV but without CAD, and the criteria for selecting control patients were as follows: (1) hospitalization in the same year, (2) comparable age stratification, and (3) same gender.

This study was conducted in accordance with the Declaration of Helsinki and approved by the Institutional Review Board (IRB) of Beijing Children’s Hospital, Capital Medical University (2020-k-021). All patients’ parents signed informed consent.

### Diagnostic criteria

The inclusion criteria for diagnosing CAEBV in this study were as follows [7, 8]: (1) persistent or recurrent IM-like symptoms for > 3 months, such as fever, liver dysfunction, lymphadenopathy, hepatosplenomegaly, hydroa vacciniforme, and hypersensitivity to mosquito bites; (2) EBV antibodies (EBV-CA and EBV-EA) were detected in tissues or peripheral blood samples or EBV-encoded small RNA (EBER) positive cells in tissues or EBV-DNA in plasma and whole blood > 10^2.5^ copies/ml; and (3) no identifiable underlying immunodeficiency disease. Patients had to fulfill all the three criteria. The T cell or NK cell type of CAEBV was mainly determined by immunohistochemistry of EBERs in fractionated CD3^+^ or CD56^+^ cells, or the copy number of EBV-DNA in fractionated CD3^+^ or CD56^+^ cells. The clonality of the EBV-infected cells was assessed by Southern blotting using EBV terminal repeats or T-cell receptor genes.

The diagnostic criteria for HLH were according to the HLH-04 criteria proposed by the International Histiocyte Society [9].

CAD was defined as an abnormal coronary dilatation (segmental or diffuse) > 1.5 times of the reference normal value either in the same artery or in other adjacent normal arteries. The normal range for Chinese children's coronary arteries was determined by referring to the published paper by Du [10]. The internal diameter of the coronary arteries was measured by transthoracic echocardiography and assessed using the z-score (http://zscore.chboston.org/).

### Therapeutic regimens [11]

(1) The first step was using an L-DEP regimen to reduce the number of EBV copies and EBV-infected T and/or NK cells. L-DEP included the following treatments: 2000 U/m^2^ of PEG-asparaginase on day 5; 25 mg/m^2^ of doxorubicin on day 1; 100 mg/m^2^ of etoposide on days 1, 8 and 15; and 15 mg/kg/day (days 1–3), 2 mg/kg/day (days 4–7) and 1 mg/kg/day (days 8–14) of methylprednisolone followed by gradual tapering the following week.

(2) In the second step, after 2 cycles of chemotherapy, patients were referred for allogeneic stem cell transplantation. The L-DEP regimen could be repeated in patients who did not receive allo-HSCT for various reasons, with a maximum of four courses.

### Statistical analysis

The results of the statistical analysis are expressed as the median (range). Statistical analysis was performed by using IBM SPSS Statistics 24 software (IBM, USA). Skewed data are presented as the median (quartile). The independent-samples t-test was used to test for differences between quantitative variables. The Kolmogorov–Smirnov test was used to verify the overall survival rate, and the log-rank test was used to compare the survival rate between different groups. *P* < 0.05 was considered a significant difference.

## Results

### General patient information

Ten patients with CAEBV combined with CAD were enrolled in this study, accounting for 8.9% (10/112) of CAEBV cases in the same period. The median age of disease onset was 6.1 (2.8–14.3) years. The ratio of males to females was 1.5:1 (Table 1). In all, 7 patients were defined as having T-cell type disease, and 3 patients were defined as having NK-cell type disease.

**Table 1:** General information

Pt	Sex	Onset age (years) (岁)	Final diagnosis	Treatment	Time of CAD recovery	Duration of follow-up (month)	Result
1	F	10.5	CAEBV	L-DEP	Non-recovered	8.0	Alive
2	M	6.3	CAEBV	Abandon	Non-recovered	12.0	Alive
3	M	12.2	CAEBV, HLH	Abandon	Non-recovered	13.0	Alive
4	F	5.8	CAEBV	Abandon	Non-recovered	27.0	Alive
5	M	14.3	CAEBV	L-DEP	18 days	28.0	Alive
6	M	3.2	CAEBV	L-DEP, HSCT	68 days	28.0	Alive
7	M	7.6	CAEBV	L-DEP, HSCT	Non-recovered	6.0	Dead
8	F	4.3	CAEBV	L-DEP, HSCT	Non-recovered	48.0	Alive
9	F	2.8	CAEBV,HLH	L-DEP	Non-recovered	8.0	Alive
10	M	5.0	CAEBV, HLH	L-DEP, HSCT	21 days	30.0	Alive

*Pt* patient, *CAEBV* chronic active Epstein–Barr virus infection, *HLH* hemophagocytic lymphohistiocytosis, *CAD* coronary artery dilatation, *L-DEP* PEG-Aspegaspargase, doxorubicin, etoposide and methylprednisolone, *time of CAD recovery* the time from discovery of CAD to recovery

### Clinical manifestations and laboratory examination

In the early stages of the disease, all patients presented with various degrees of hepatomegaly, eight patients had different degrees of splenomegaly, seven patients had fever, and the median duration of fever was 1 (0.5–6) months. Five patients developed skin rashes, which were mainly red papules or maculopapules without itching, occurring predominantly on the torso. Five patients had lymphadenopathy, 4 of whom had this located in the cervical region and 1 of whom had this located in the bilateral axillae. Before admission, all the children had no symptoms of Kawasaki disease (KD), such as red eyes, red lips, red bayberry tongue, limb swelling, or molting. Two patients were admitted to the hospital with "incomplete Kawasaki disease" (cases 6 and 7). The other 8 patients were admitted to the hospital for fever or hepatosplenomegaly.

After admission, laboratory examinations showed that 7 patients (cases 1, 3, 5, 6, 7, 9 and 10) had varying degrees of hematocytopenia. Five patients (cases 2, 3, 5, 9 and 10) had high triglyceride levels (> 3 mmol/L), 2 patients had (cases 1 and 9) had low NK cell activity (< 15.11%), and 2 patients (cases 9 and 10) had a hemophagocytic phenomenon on a bone marrow smear. The EBV antibody spectrum of all children indicated reactivation of a previous infection. All ten patients tested positive for EBER. Biopsy was performed of the bone marrow (8 cases), lymph nodes (2 cases) and liver (1 case). Serological and PCR tests for other pathogens (such as herpes zoster virus, hepatitis B virus, cytomegalovirus, bacteria and parasites) were negative. All exon genetic examinations and bone marrow cell flow cytometry evaluations were normal. The other laboratory examination results of the 10 patients are summarized in Table 2.

**Table 2:** Laboratory data at diagnosis

Pt	WBC (× 10^9^/L)	Hb (g/L)	PLT (× 10^9^/L)	ALT (U/L)	AST (U/L)	CK-MB (U/L)	EBV-DNA (whole blood) (Copies/mL)	EBV-DNA (plasma) (Copies/mL)	sCD25 (ng/L)	SF (ug/L)	IL-6 (ng/L)	IL-10 (ng/L)	TNF-α (ng/L)
1	2.45	108	144	1928.0	1806.0	24	1.56 × 10^7^	5.00 × 10^3^	1830.0	216.0	13.95	7.06	6.57
2	6.01	121	137	524.0	584.1	20	1.60 × 10^7^	2.94 × 10^4^	8922.0	296.8	14.53	6.03	1.16
3	1.49	73	56	240.2	108.0	32	6.37 × 10^6^	6.75 × 10^3^	4574.0	89.3	12.45	10.15	2.59
4	6.41	122	422	68.9	141.6	22	7.70 × 10^6^	2.08 × 10^3^	1504.0	108.6	143.85	2.60	113.62
5	2.57	102	201	464.8	331.8	32	3.50 × 10^5^	3.17 × 10^3^	2541.0	509.4	11.77	3.23	0.00
6	7.87	98	118	221.2	368.5	14	1.15 × 10^7^	3.77 × 10^5^	23,684.0	888.0	19.44	18.00	3.60
7	5.19	111	137	38.4	26.2	19	1.9 × 10^5^	1.30 × 10^5^	3847.6	64.7	2500.00	1.79	525.62
8	9.91	131	270	22.8	12.2	10	3.96 × 10^7^	1.54 × 10^3^	3849.2	27.8	6.46	3.22	0.31
9	4.52	94	110	37.1	29.4	18	1.20 × 10^7^	5.17 × 10^4^	2987.0	67.5	4.98	4.25	2.73
10	4.87	104	30	876.0	103.4	33	1.44 × 10^7^	1.76 × 10^6^	8519.0	91,893.2	76.98	270.01	0.71

*Pt* patient, *WBC* white blood cell, *Hb* hemoglobin, *PLT* platelet, *ALT* alanine transaminase, *AST* aspartate transaminase, *CK-MB* creatine kinase MB, *EBV* Epstein–Barr virus, *CA* capsid antigen, *EA* early antigen, *NA* nuclear antigen

### Cardiac complications

**Fig. 1:**
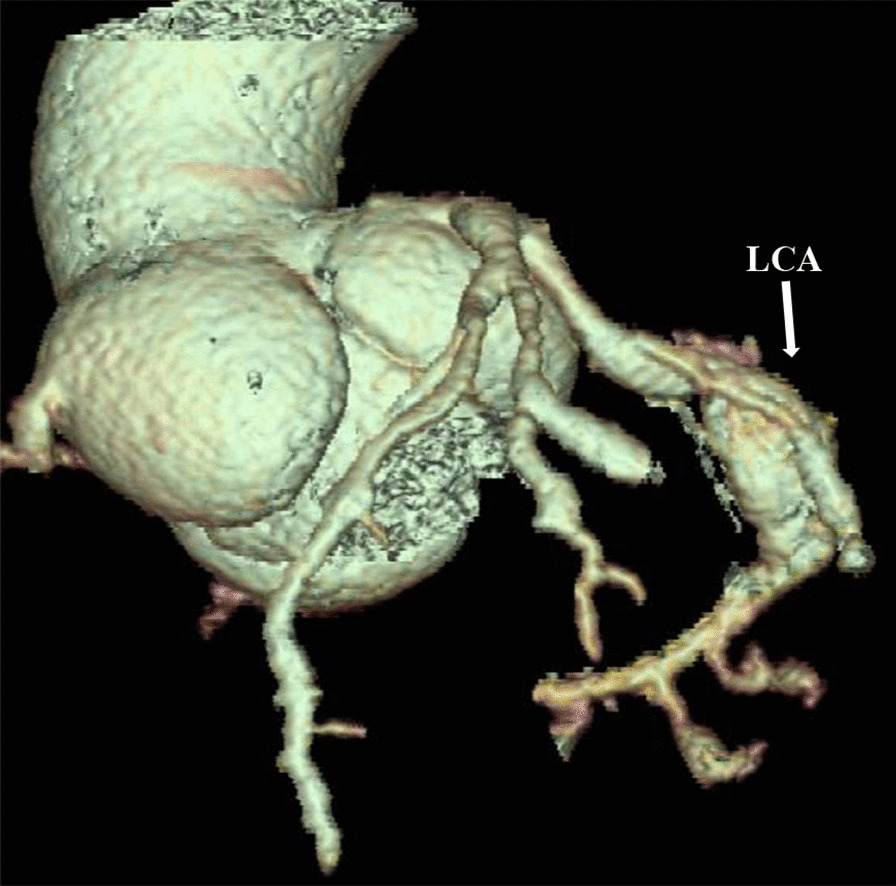
Three-dimensional CT reconstruction of the aorta showing dilation of the right coronary artery and aneurysm of the left coronary artery (Patient 10)

The date of echocardiography of the coronary arteries is shown in Table 3. Of the 10 patients, 8 had bilateral CAD (80%), and 2 had left CAD (20%) (Fig. 1). The z-score of left coronary artery was 4.13 (2.76–9.28), and that of the right coronary artery was 5.28 (0.99–9.63) No obvious abnormal blood flow signal was found in the coronary artery. ECG showed an abnormal ST-T voltage in 1 patient (case 3), a prolonged PR interval (0.19 s) in 3 patients (cases 2, 5, and 7) and T-wave inversion in 1 patient (case 1).

**Table 3:** Coronary artery lesions in patients with chronic active Epstein–Barr virus (EBV) infection

Pt	Affected branch	LCA Diameter (mm)				RCA Diameter (mm)				Body surface area (m^2^)	Z score (left/right)	Shape of vessel
		Main	Bifurcation	Anterior descending branch	Circumflex	Orifices	Proximal	Middle	Distal			
1	LCA/RCA	4.9–5.7	6.4	3.0–3.5	3.4	5.0	5.1–5.9	5.3–6.1	3.5	1.030	5.91/8.87	Aneurysms
2	LCA/RCA	3.5	–	3.2	2.7	4.2	3.9	3.4	2.5	0.605	2.84/6.01	Aneurysm
3	LCA	4.0–4.5	–	2.7	2.7	–	3.0	–	2.1	1.210	2.76/0.99	Dilated
4	LCA/RCA	4.6	-	3.0	3.3	–	2.9–3.5	2.2	2.2	0.748	4.79/4.02	Aneurysms
5	LCA/RCA	4.8	–	3.6	3.1	–	3.8	2.6	2.6	1.250	3.23/2.77	Aneurysm
6	LCA/RCA	3.9–5.8	4.5	3.6	2.2	–	2.7–4.9	3.2–3.7	2.5–3.3	0.525	9.28/9.63	Aneurysms
7	LCA/RCA	5.2–5.6	6.2	3.5	4.4–5.7	5.4	5.0–5.8	5.3	2.7	0.870	6.48/9.59	Aneurysms
8	LCA/RCA	4.5	–	2.8	2.3	4.9	5.7	2.3–3.3	2.3–3.3	0.835	4.14/9.56	Aneurysms
9	LCA/RCA	3.4	–	2.2	2	2.9	3.3	4.9–5.9	2.3	0.556	2.85/4.54	Aneurysm
10	LCA	4.2	–	3.6	2.6	–	2.8	2.5	1.9	0.695	4.11/2.30	Aneurysm

*Pt* patient, *LCA* left coronary artery, *RCA* right coronary artery, – test not performed

Echocardiography showed a normal left ventricular ejection fraction ranging from 62 to 79% and a left ventricular shortening fraction ranging from 32 to 46%. Three patients had mild mitral and aortic valve regurgitation (cases 1, 3, and 8). 3 patients had mild tricuspid regurgitation (cases 3, 5, and 7). Echocardiography and chest x-rays showed mild pericardial effusion in 4 patients (cases 3, 5, 6, and 8). No cardiac biopsies were performed.

### Diagnosis, treatment, and prognosis

All the children met the diagnostic criteria for CAEBV, and 3 of them were complicated with HLH (cases 3, 9, and 10). Of the 10 patients, 3 patients discontinued treatment due to their own reasons (cases 2, 3, and 4) (mostly due to financial reasons). The remaining 7 patients received the L-DEP protocol. Allo-HSCT was performed for 4 patients (cases 6, 7, 8, and 10). All patients underwent related-donor haploidentical stem cell transplantation. The other 3 patients were still on chemotherapy before HSCT (case 1, 5, 9).

The last follow-up was July 1, 2020, and the median follow-up time was 20 (6–48) months. For 3 patients (cases 5, 6, and 10) who underwent treatment, the lumen of the coronary arteries regressed to normal size, and the echogenicity of the arterial walls reduced normally. The time from discovery of CAD to a return to normal was 21 (18–68) days, including 2 patients with CAD who recovered after chemotherapy and 1 patient who recovered after HSCT. One patient (case 7) died of a severe infection related to HSCT. There were no significant changes in the coronary arteries of the remaining 3 patients. At the time of the last patient follow-up record, no patients died of bleeding, thrombus formation and/or pericardial tamponade caused by ruptured coronary artery aneurysms (Table 1).

### Comparison of CAEBV patients with and without CAD

**Fig. 2:**
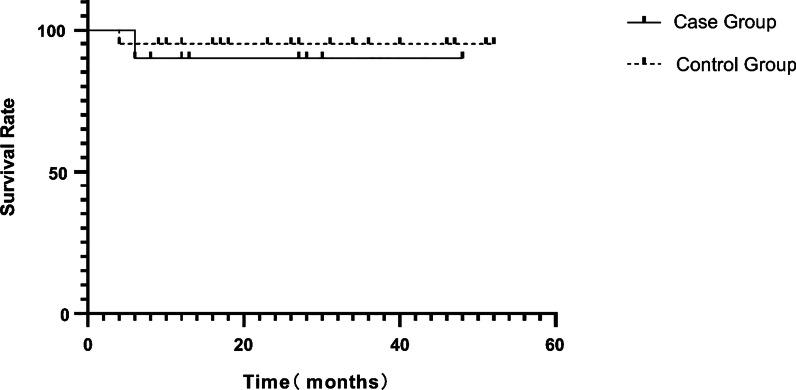
There was no significant difference in the rate of survival between the two groups (90.0% vs. 95.0%, log-rank test, *P* = 0.631)

We compared the characteristics of CAEBV patients with and without CAD (Table 4). LDH, SF, TNF-α and IL-10 levels were relatively higher in patients with CAEBV with CAD than in those with CAEBV without CAD (*P* = 0.009, 0.008, 0.026 and 0.030, respectively). The incidence of lymphadenopathy, hepatosplenomegaly and the EBV copy number did not differ between patients with and without CAD (*P* = 0.625, 4.565, 0.093, 0.284 and 0.992, respectively). There was no significant difference in the rate of survival between the two groups (90.0% vs. 95.0%, log-rank test, *P* = 0.631) (Fig. 2).

**Table 4:** Comparison of CAEBV with and without CAD

	Case group (n = 10)	Control group (n = 20)	*P* Value
Course of disease (d)	70.57	86.31	0.693
Lymphadenopathy	5	13	0.625
Hepatomegaly	10	13	4.565
Splenomegaly	8	15	0.093
WBC (× 10^9^/L)	5.03 (1.49–9.91)	4.82 (1.13–12.98)	0.205
ANC (× 10^9^/L)	2.66 (0.42–5.11)	1.74 (0.36–7.89)	0.473
Hb (g/L)	106.00 (73.00–131.00)	105.00 (21.00–341.00)	1.000
PLT (× 10^9^/L)	137.00 (30.00–422.00)	187.00 (21.00–341.00)	0.890
ALT (U/L)	230. 70 (22.80–1928.00)	109.85 (19.30–695.00)	0.090
AST (U/L)	124.80 (12.20–1806.00)	46.50 (9.90–662.90)	0.450
TG (mmol/L)	2.91 (1.20–5.24)	1.71 (0.66–5.71)	0.797
Fib (g/L)	2.12 (1.35–3.11)	1.29 (0.90–3.50)	0.890
CK-MB (U/L)	21.00 (10.00–33.00)	17.50 (6.00–45.00)	0.598
LDH (U/L)	433.500 (226.00–9934.00)	219.50 (201.00–1393.00)	0.009
CD4 + /CD8 +	0.75 (0.02–2.03)	2.31 (0.73–7.95)	0.049
SF (ug/L)	162.3 (27.80–91,893.20)	156.05 (24.70–17,043.00)	0.008
sCD25 (ng/L)	3848.40 (1504.00–23,684.00)	9282.20 (1500.00–40,623.00)	0.080
EBV (plasma) (Copies/mL)	1.81 (0.15–176.00) × 10^4^	2.65 (0.15–755.00) × 10^4^	0.284
EBV (whole blood) (Copies/mL)	1.18 (0.02–3.96) × 10^7^	0.17 (0.016–3.97) × 10^7^	0.992
NK cell activity (%)	15.30 (12.84–20.52)	14.52 (10.82–50.80)	0.282
IFN-γ (ng/L)	13.68 (3.41–790.41)	76.29 (0.00–769.91)	0.585
TNF-α (ng/L)	2.66 (0.00–525.62)	2.37 (0.00–244.33)	0.026
IL-6 (ng/L)	14.24 (4.98–2500.00)	31.64 (0.00–2500.00)	0.384
IL-10 (ng/L)	5.14 (1.79–270.01)	6.14 (1.93–75.72)	0.030

*WBC* white blood cell, *ANC* neutrophil, *Hb* hemoglobin, *PLT* platelet, *ALT*: alanine transaminase, *AST* aspartate transaminase, *TG* triglyceride, *Fib* fibrinogen, *CK-MB* creatine kinase MB, *LDH* lactic dehydrogenase, *SF* serum ferritin, *EBV* Epstein–Barr virus, *IFN-γ* Interferon-γ, *TNF-α* tumor necrosis factor-α, *IL* interleukin

## Discussion

As a γ-herpesvirus, EBV is mainly transmitted through saliva. Reports of EBV infection with CAD are relatively rare. To date, only 25 cases have been reported. Among them, 16 cases were IM combined with CAD [12, 13], 3 cases were EBV-HLH combined with CAD [14–16], and 6 cases were CAEBV combined with CAD [4, 5]. All 25 patients were children and were equally split between males and females (male–female ratio of 1.08). Of the 25 patients, 4 had bilateral CAD (16%), 14 had right CAD (56%), and 7 had left CAD (28%). The majority IM patients with CAD had the right coronary artery affected (81.2%); however, in EBV-HLH and CAEBV patients, the left coronary artery was affected (100%). In this study, we described the characteristics of 10 CAEBV patients with CAD and found that all cases of CAD involved the left coronary artery. Consistent with previous reports, there was no gender difference or specificity in the age of onset. Regarding patients infected with EBV combined with CAD, the cardiac structure might be abnormal, but the cardiac function could be normal.

The mechanism of EBV infection leading to CAD is unclear, but may be related to the following factors. EBV antigenic determinants on the cell surface of cytotoxic T cells (CTLs), such as latent membrane protein (LMP)-1 [17], could be changed when CTLs are infected by EBV. LMP-1 can significantly increase the production of vascular endothelial growth factor (VEGF) in vivo [18]. VEGF can increase the permeability of retrocapillary veins and venules by inducing the production of related zymogen activators, resulting in vascular wall destruction and vascular involvement [19]. EBV can also activate the Janus kinase-signal transducer and transcriptional activator (JAK-STAT) pathway, which leads to the transcription of angiogenic genes. This can promote cell migration, invasion and angiogenesis in an autocrine and paracrine manner. However, excessive growth and persistent stimulation related to pathological angiogenesis lead to basement membrane defects and uneven pericyte coverage of these vessels [20]. In addition, Dogan [21] and Ariza [22] found that EBV can produce deoxyuridine triphosphatase in the process of replication, which can increase the level of interleukin (IL)-6 in vivo, and IL-6 can lead to vascular endothelial damage, resulting in CAD. Tumor necrosis factor (TNF)-α binds to the corresponding receptors on LMP-1, which leads to the activation of intracellular PKC and PKA; they can then bind to and active nuclear factor (NF)-κB. Studies have demonstrated that NF-κB can lead to the degradation of extracellular matrix in the arterial wall, promote an inflammatory response, and accelerate the occurrence of hemangioma [23].

This retrospective observational study showed several important findings. The levels of TNF-α and IL-10 of CAEBV were relatively higher in patients with CAD than in those without. As mentioned above, TNF-α and IL-6 can lead to vascular endothelial damage, resulting in CAD. CAEBV patients with high levels of TNF-α and IL-6 should be paid more attention to in cases of secondary CAD. At the same time, our study also found that CD4+ /CD8+ levels were lower in CAEBV patients with CAD, suggesting that a disorder of T-cell immune function may also be one of the main causes of CAD, especially when CD4+ T cells decrease or CD8+ T cells increase. In addition, patients with high ferritin in the early stage of CAEBV should also be monitored for the possibility of developing secondary CAD.

Coronary artery dilatation, especially coronary artery aneurysm, is most common in KD in childhood, and it is one of the characteristics of KD [24]. EBV infection combined with CAD or with KD has many similarities in clinical manifestations, such as fever, cervical lymphadenopathy, and rash, which makes these diseases difficult to distinguish. Of the 10 patients, 2 (20%) were misdiagnosed with atypical KD at the early stage of the disorder. However, these patients did not have conjunctival congestion, bayberry tongue, fingertip scleroderma, molting, and elevated platelets, suggesting that the diagnosis of KD was erroneous. The treatment and prognosis of CAEBV combined with CAD versus KD are significantly different; thus, differential diagnosis of these two diseases is necessary. For patients with a fever lasting more than 2 weeks accompanied by hepatosplenomegaly, elevated transaminase and coronary artery dilatation without other typical manifestations of KD, the possibility of EBV infection should be highly suspected. The detection of EBV antibody and EBV-DNA should be completed as soon as possible, and bone marrow or lymph node biopsy should be performed if necessary. CAEBV patients have a poor prognosis, and some of them have rapid progression. Therefore, once the diagnosis of CAEBV is confirmed, chemotherapy and subsequent HSCT should be performed as soon as possible [6].

The incidence of EBV infection with CAD is low. Previous studies have found that the incidence of IM with CAD is 4.4% [12]. However, no incidence of EBV-HLH with CAD has been reported. This study found that the incidence of CAEBV complicated with CAD was 8.9%. Fourteen previous cases (56%) returned to normal, including 13 cases of IM combined with CAD and 1 case of CAEBV combined with CAD. In this study, 3 cases of coronary artery dilatation returned to normal, accounting for 30.0% of cases of CAEBV with CAD, and no patients died of CAD-related complications. EBV infection combined with CAD does not affect the prognosis of the primary disease; therefore, controlling the primary disease and eliminating EBV infection are the key points of treatment. Previous studies have found that if EBV infection in the body cannot be cleared or effectively controlled, it is difficult for CAD to resolve [4]. CAD should be examined by echocardiography regularly to monitor for dynamic changes. Doctors should also be alert as to whether CAD is complicated with abnormal cardiac function.

## Conclusions

The incidence of CAEBV with CAD was low. CAEBV with CAD did not influence the prognosis. Patients who had high LDH, serum ferritin, TNF-α, and IL-10 levels early in their illness were more likely to develop CAD. There were some shortcomings in this study: the number of patients was small, and the mechanism of CAD secondary to EBV infection still needs to be further studied.

## Data Availability

The data that support the findings of this study are available on request from the corresponding author.

## Abbreviations

EBV: Epstein–Barr virus
IM: Infectious mononucleosis
HLH: Hemophagocytic lymphohistiocytosis
CAEBV: Chronic active Epstein–Barr virus infection
CAD: Coronary artery dilatation
EBER: Epstein–Barr virus-encoded small RNA
L-DEP: PEG-Aspegaspargase, doxorubicin, etoposide and methylprednisolone
LVEF: Left ventricular ejection fraction
LVSF: Left ventricular shortening fraction
CTL: Cytotoxic T cell
LMP: Latent membrane protein
VEGF: Vascular endothelial growth factor
JAK-STAT: Janus kinase-signal transducer and activator of transcriptions
IL: Interleukin
NF-κB: Nuclear factor κB
HSCT: Hematopoietic stem cell transplantation
KD: Kawasaki disease
